# Nonsurgical Treatment for Localized Hepatocellular Carcinoma

**DOI:** 10.1007/s11912-013-0373-x

**Published:** 2014-02-02

**Authors:** Andrew S. Kennedy, Bruno Sangro

**Affiliations:** 1 Radiation Oncology Research, Sarah Cannon Research Institute, 3322 West End Avenue, Suite 800, Nashville, TN 37203 USA; 2 Department of Biomedical Engineering, North Carolina State University Raleigh, Raleigh, NC USA; 3 Department of Mechanical and Aerospace Engineering, North Carolina State University Raleigh, Raleigh, NC USA; 4 Liver Unit, Clinica Universidad de Navarra, Pamplona, Spain; 5 Centro de Investigacion Biomedica en Red de Enfermedades Hepaticas y Digestivas (CIBEREHD), Pamplona, Spain

**Keywords:** Yttrium, SIRT, Selective internal radiation therapy, TACE, Transarterial chemoembolization, TAE, Transarterial embolization, External beam radiotherapy, Proton, SBRT, Stereotactic body radiotherapy, Radioembolization, Microspheres, Oncology, Gastrointestinal cancer

## Abstract

The most common non-surgical approaches for the treatment of localized hepatocellular carcinoma remain hepatic artery-delivered particles laden with chemotherapy (TACE), or radioactive microparticles (TARE). External beam radiotherapy has been an effective option in many parts of the world for selected HCC patients, but now has an expanded role with stereotactic and proton beam technologies. This review focuses on existing evidence and current guidance for utilizing these modalities for localized, but unresectable, non-transplantable HCC patients.x

## Introduction

The incidence and mortality of hepatocellular carcinoma (HCC) continues to increase worldwide and is the third leading cause of global cancer deaths [1]. In the USA, HCC mortality has been rising steadily for more than two decades with no plateau seen [2]. A dismal overall 5-year survival rate of only 5 % is achieved, and more than 70 % of patients present with advanced disease that cannot be approached with curative intent—i.e., transplantation, resection, or tumors <3 cm that can be ablated [3].

The focus of this review is limited to treatment of the largest group of HCC patients seen worldwide—the approximately 70 % who do not have curative options due to tumor size, number and/or location, and do not have metastatic disease. The most commonly employed approaches for control of HCC for the majority of patients therefore fall into two categories, which will be presented in depth: hepatic artery delivered therapies, and externally delivered radiation therapy.

## Intra-Arterial Therapies

When related to the treatment of unresectable HCC, intra-arterial therapies are a group of treatments where therapeutic and/or embolic agents are intra-arterially directed to target tumors. The unique double irrigation of the liver (through the portal vein and the hepatic artery) and the predominantly arterial irrigation of liver tumors, particularly primary liver cancer, are the basis for intra-arterial therapies. The most commonly used techniques are transarterial chemoembolization (TACE) with or without drug-eluting beads (DEB) and transarterial radioembolization (TARE), also called selective internal radiation therapy. TACE combines local drug delivery with concurrent tumor-feeding artery embolization. TARE is a form of brachytherapy in which intra-arterially injected microspheres loaded with yttrium 90 (^90^Y) serve as sealed sources for internal radiation purposes.

## Transarterial Chemoembolization

TACE embraces different procedures that share two different aims: (1) to increase the exposure of tumor cells to cytotoxic agents, and (2) to induce ischemic necrosis. This is usually accomplished by the sequential intra-arterial injection of chemotherapeutic agents and embolizing particles. The wide variety of drug vehicles, cytotoxic agents, and embolizing particles has introduced numerous variations worldwide and no standard protocol has been uniformly adopted. Centers have different choices regarding type and/or dose of the anticancer agents used, use of lipiodol as a vehicle, embolizing material, selectivity of catheter positioning, embolization end-points, and schedule and/or interval of retreatment. Although conventional TACE with administration of an anticancer-in-lipiodol emulsion followed by embolic agents has been the most popular technique, bland embolization is still preferred in some centers, and TACE with drug eluting particles (DEB-TACE) have replaced conventional regimens in others.

### Conventional Tace

In TACE of HCC, single-agent doxorubicin is most commonly used in Europe and Japan, while the combination of mitomycin C, doxorubicin, and cisplatin is more popular in the United States. Single or combination agents are typically emulsified in lipiodol, an oily contrast agent believed to increase intratumoral retention of the cytotoxic agent. The iodized oil persists selectively in the tumor for a few weeks or months because of hemodynamic differences between hypervascular hepatic tumors and liver parenchyma, and presumably because of the absence of Kupffer cells in tumors. This is followed by embolization of the target vessels with agents such as gelfoam (heterogeneous in size), or the more recently calibrated polyvinyl alcohol, or acrylic copolymer gelatin particles. The use of calibrated particles is increasing worldwide since they can be chosen by size according to the target vessel [4]. Vessel occlusion following injection of this mixture results in lower peak plasma concentrations and increased drug retention inside tumors. However, the therapeutic benefit obtained from adding a cytotoxic agent or lipiodol to bland embolization was challenged by the results of two clinical trials conducted in the 1990s [5, 6] and two meta-analyses [7, 8] suggesting that the antitumor effect is mainly driven by ischemia.

The evidences that support the use of TACE for palliative treatment of unresectable HCC are randomized controlled trials [9, 10] in selected patients with preserved liver function. In the Spanish trial [9], patients with preserved liver function and free from main portal vein thrombosis (PVT) were treated with fixed interval chemoembolization or embolization, or best supportive care. The 2-year survival rate after TACE was 63 % compared to 27 % in the untreated patients (P = 0.009). In the Hong-Kong trial [10], patients with lobar or branch PVT were included provided their liver function was preserved, and 2-year survival rate of 31 % was again superior to the 11 % observed in the control group (P = 0.002). Three other clinical trials had failed to show a superiority of TACE compared to best supportive care or inactive treatments (such as tamoxifen) in terms of survival [11–13]. Three meta-analyses [7, 8, 14] have subsequently confirmed that TACE improves survival of patients, and TACE has been established as the standard of care for patients in the intermediate stage of the Barcelona Clinic Liver Cancer (BCLC) staging system, i.e., those with multinodular HCC, relatively preserved liver function, absence of cancer-related symptoms, and no evidence of vascular invasion or extrahepatic spread [15].

However, it should be noted that approximately one-half of patients recruited in the two positive trials had one or two tumor nodules and were likely early-stage patients in which ablation was deemed unfeasible. In fact, the range of patients treated by TACE in clinical practice has exceeded, by much greater margins, intermediate-stage disease. As a result, reported survivals are rather heterogeneous, ranging from 53 to 90 % at 1 year, 11 to 67 % at 2 years and 8 to 26 % at 5 years [16–24]. Median survival averages 16 months, even in contemporary series with unrestricted patient selection [25–27]. By stage, reported median survival ranges from 16 to 45 months in the early BCLC A stage, from 15.6 to 18.2 months in intermediate BCLC B stage, and from 6.8 to 13.6 in the advanced BCLC C stage. Prognosis after TACE largely depends on liver function [20, 22, 23, 28–30], tumor burden [16, 22, 23, 31] presence of portal vein invasion [10, 16, 17], and most importantly, response to treatment [9, 32]. TACE is generally contraindicated in patients with PVT, since occlusion of arterial blood flow may induce liver failure. However, superselective TACE may be safe in selected patients with segmental branch invasion [33, 34].

TACE is generally a safe procedure, although it is frequently followed by side effects that can occasionally be severe. The most common is post-embolization syndrome, consisting of nausea, abdominal pain, and fever, which occurs in more than 40 % of patients, but tends to be mild and transient. A variable proportion of patients (20–45 %) show a transient decline in liver function after TACE and acute liver decompensation (appearance of ascites, encephalopathy or jaundice), reported in 0.1–3 % of cases [35, 36]. Biliary and gastrointestinal tract complications have been reported in 2–10 % [37, 38] and 1–5 % [34] of patients, respectively. Mortality rates range widely, from 0.003 to 10 % in the different series [7, 16, 39], again reflecting differences in the target population and TACE regimen. Liver functional reserve is key to an optimal selection and patients should be in the Child–Pugh A class or B7 without ascites. A recent expert panel consensus has recommended a series of absolute and relative contraindications for the treatment of patients in the intermediate stage [40] that also apply to the advanced stage.

### Tace Using Drug-Eluting Particles

The concept of DEB-TACE is to intra-arterially inject embolizing particles that have been loaded in vitro with cytotoxic agents and slowly release them into the tumor environment. They are composed of either a sulfonate-modified poly(vinyl alcohol) hydrogel (DC-Beads, Biocompatibles, Surrey, UK) or a sodium acrylate and vinyl alcohol copolymer (HepaSphere, BioSphere Medical, Inc., Rockland, MA, USA). For the treatment of HCC, these particles can be loaded with doxorubicin [41]. In a large, international, randomized trial that compared conventional TACE with DEB-TACE using DC Beads, the primary endpoints (superiority of DEB-TACE in achieving objective tumor response at 6 months and in producing fewer treatment-related serious adverse events in the first 30 days) were not met [42]. Tumor response rates were 52 vs. 44 % and time to progression was 7.1 vs. 6.4 months for DEB-TACE and conventional TACE, respectively. Most likely, the expectations in the control arm were based on early reports on conventional TACE and were thus overly pessimistic. A similar 6-month response rate of 51 % was reported for HepaSphere in multicenter study [43]. Furthermore, a prospective, randomized comparison of DEB-TACE and bland embolization using the same unloaded particles showed that despite producing a significantly better response rate at 9 months (55 % vs. 31 %), 12-month survival was similar (85.3 % vs. 86 %) [44]. Even if it does not improve survival over conventional TACE, DEB-TACE has provided a way of performing TACE in a much more standardized way and has shown that when the optimal patients are selected, the beneficial effect of TACE can indeed challenge that of percutaneous ablation. Recent reports on nearly 300 patients in early and intermediate stages from two experienced centers show 3-year and 5-year survival rates of 62–66 % and 22–38 %, respectively [45]. DEB-TACE is generally well-tolerated. Major complications occurred in 4.1 % of patients in a Greek series [44] including liver abscess, cholecystitis, pleural effusion, and three cases (1.6 %) of irreversible liver failure, and in 10 % of patients in a Spanish series [41], reporting a treatment-related death rate of 0.96 %.

## Transarterial Radioembolization

TARE comprises those procedures in which intra-arterially injected radioactive microspheres are used for internal radiation treatment [46]. There are two types of commercially available radioactive microspheres made of glass (TheraSphere; MDS Nordion, Ottawa, Ontario, Canada) and resin (SIR-Spheres; Sirtex Medical Limited, Sydney, Australia), although they both use ^90^Y as the radiation-emitting isotope. ^90^Y is a pure beta emitter with half-life of 64.2 hours (94 % of the energy is emitted in the first 11 days) and average tissue penetration of 2.5 mm (isolation for radiation protection is not needed after implantation). Due to their small size (25–45 microns), they produce no significant ischemic effect as opposed to the greater than 100-micron particles used in TACE. To avoid misplacement of particles in extrahepatic territories, a thorough angiographic evaluation is performed 1–2 weeks prior to treatment to detect and eventually occlude aberrant vessels arising from hepatic arteries that may feed the gastrointestinal tract, and to measure the hepatopulmonary shunting using technetium-99 m-labeled macroaggregated albumin. Patients can only be considered for TARE provided their liver function is preserved (serum total bilirubin <2 mg/dL) and they have no ascites or hepatic encephalopathy.

No randomized, controlled trial comparing TARE with others therapies has been published yet, although good level 2 evidence can be compiled from large, well-characterized cohort series published over the last 5 years [40, 47–51]. By and large, TARE has been mainly used to treat unresectable patients considered suboptimal candidates for TACE (those in advanced stage due to symptoms or PVT or those in intermediate stage with very large tumors or extensive bilobar involvement [40]. In these poor TACE candidates, a case-control study indicated that TARE might improve survival compared to experimental therapies or best supportive care (16 vs. 8 months, p < 0.05) [52]. When analyzed by tumor stage, intermediate stage patients treated by TARE reach a median survival of 16–18 months [49–51] that compares well with the median survival achieved by TACE. Broadly equivalent survivals have also been reported in retrospective analyses from single institutions although treatment selection and lead-time biases should be considered. For patients in the intermediate stage who fail to respond to TACE, remaining treatment options include the antiangiogenic/antiproliferative targeted agent sorafenib or TARE. The sorafenib HCC Assessment Randomized Protocol (SHARP) was the pivotal phase III, randomized, controlled trial that proved that systemic agent sorafenib prolonged the survival of HCC patients [53]. The target population was patients not amenable for TACE, including those with advanced-stage HCC and those in the intermediate stage that had progressed or were considered poor TACE candidates. In a subset analysis of the SHARP trial, survival in patients failing TACE was 11.9 and 9.9 months, respectively, for the sorafenib and placebo-treatment arms [54]. By comparison, survival was 11.4 months for a subset of usual candidates for TARE matching the SHARP criteria, and 15.4 months in BCLC B patients failing TACE [51].

Sorafenib is the mainstay for treating advanced HCC, defined by the presence of vascular invasion, extrahepatic disease or poor performance status in a patient with at least partially preserved liver function. As TARE has no macroembolic effect [55]. it can be safely applied to patients with PVT, and can offer a median survival in the range of 6–13 months [47, 49–51], very similar to the 6.5–10.7 months reported in the phase III clinical trials of sorafenib in the same group of patients [24, 53]. Furthermore, in patients with only branch or segmental PVT, survival increases to 10–14 months [49, 56, 57]. Due to this growing body of level 2 evidence, TARE has found a place in the guidelines adopted by the European Society for Medical Oncology (ESMO), the European Society of Digestive Oncology (ESDO), and the National Comprehensive Cancer Network (NCCN), albeit not in the guidelines of the European Association for the Study of the Liver (EASL), the European Organization for Research and Treatment of Cancer (EORTC), or the American Association for the Study of the Liver Diseases (AASLD).

The indications described above have been adopted as standard in many referral centers. Other interesting indications that are still considered investigational target the population with less advanced tumors [58]. TARE can induce complete necrosis in small (< 3 cm) tumors as shown in the analyses of 35 explanted livers [59]. In patients with inoperable early stage HCC, median time to progression as long as 25.1 months (95 % CI 8–27 months) have been reported [60] and this may provide a rationale for its use as a bridge to liver transplantation in an attempt to avoid dropping off the waiting list [61]. The potential to induce intense tumor responses has allowed TARE to be used as a downstaging therapy, to reduce tumor burden within acceptable limits for liver transplantation, to render non-operable patients operable, or to simplify surgery. Downsizing from UNOS T3 to T2 was achieved more frequently with TARE than with TACE (58 % vs. 31 % p = 0.023) [62]. Furthermore, atrophy of the irradiated lobe after TARE and contralateral lobe hypertrophy as a result of the injection of a high activity of ^90^Y in a lobar hepatic artery, known as “radiation lobectomy,” may be valuable in itself and contribute to resectability [63]. In a smaller group of 21 UNOS T3 stage patients, 29 % were downstaged and underwent surgical resection or liver transplantation, with a 3-year survival rate of 75 % [64], which is comparable with the survival in patients with early-stage disease who are treated radically at the time of diagnosis.

TARE is generally well-tolerated and a post-embolization syndrome like the one that appears after TACE is not common. Rare complications resulting from the irradiation of non-tumoral tissues include pneumonitis [65], cholecystitis [66], gastrointestinal ulcerations [67], and liver damage. Liver toxicity is the most challenging adverse event in HCC patients, as the majority of these tumors arise in cirrhotic livers, with some degree of reduced functional reserve. A variable incidence of liver decompensation including ascites (0–18 %) or encephalopathy (0–4 %) has been reported [47, 57, 66, 68–70]. The incidence of radioembolization-induced liver disease (characterized by jaundice and ascites appearing 4–8 weeks after TARE) in cirrhotic patients was 9.3 % in the largest series so far reported [71].

## Combinations with Systemic Agents

Tumor hypoxia intentionally caused by TACE can induce upregulation of circulating vascular endothelial growth factor (VEGF) [58], which is essential for HCC growth, invasion, and metastasis. Recent studies have reported a significant association between VEGF upregulation after TACE and poor prognosis [72, 73]. Therefore, adjuvant or concurrent use of an anti-angiogenic agent may be helpful for HCC patients who are treated with TACE. TACE plus an anti-angiogenic agent, such as sorafenib, could potentially provide complementary inhibition of angiogenic factors and tumor growth [74], and several clinical trials are currently evaluating this combined effect on the outcome of patients with unresectable HCC. The results available thus far are somewhat disappointing. Time to progression among patients with >25 % tumor necrosis or shrinkage at 1–3 months following one or two TACE sessions that received sorafenib was not better than that of patients receiving placebo (5.4 months vs. 3.7 months, respectively; p = 0.25) [75]. When continuous sorafenib or placebo was given concurrently with DEB-TACE, safety was not an issue [76] and the hazard ratio for time to progression was 0.797 in favor of sorafenib (95 % CI 0.588–1.080; p = 0.072), but overall survival was comparable [77]. On the other hand, the addition of TARE to sorafenib for intermediate- and advanced-stage patients is being explored in the randomized, controlled SORAMIC trial (NTC 01126645). The interim analysis of the first 40 patients in the combination arm has provided further confirmation of its safety [78], and analysis of survival, which is the primary endpoint, is eagerly awaited.

## External Beam Radiation Therapy

Radiotherapy for HCC is only a recent option made possible with technological advances in EBRT [79, 80] and commercially available radioactive microsphere products. The liver architecture consists of a parallel unit structure, which has specific tolerance parameters regarding ionizing radiation. Radiation therapy to liver tumors is limited by the relative radiosensitivity of the sinusoid endothelium, compared to significantly higher required doses of radiation to confidently destroy hepatocellular carcinoma cells [81]. Various clinical and mathematical models have been developed in the past two decades to understand and predict the risk of organ damage to radiation total doses, and dose per fraction schedules. These normal tissue complication probability (NTCP) models are based on observed complications after radiotherapy in a specific organ, with known daily and total dose data and specific clinical outcomes measured [82, 83]. For hepatic radiation NTCP models, only external beam radiation with photons (not protons, carbon ions, ^90^Y, or ^131^I) has been explored. The most commonly accepted endpoint in hepatic NTCP models is radiation-induced liver disease (RILD), classically reported as TD5/5 and TD50/5. This translates into the total dose of photon radiation, typically to the whole liver, which creates a 5 % rate of RILD by 5 years post-radiation, and a 50 % rate respectively [84, 85].

## External Beam Radiotherapy for Hepatocellular Carcinoma (HCC)

Technology advances in radiation oncology in the past 20 years have rapidly expanded radiotherapy accuracy and precision to all parts of the body. Three-dimensional conformal radiotherapy (3DCRT) and intensity-modulated radiotherapy (IMRT) have been mainstays of advanced treatment delivery using computed tomography (CT)-based datasets to target tumors while sparing normal surrounding tissues. In the past decade, a specialized form of 3DCRT, which delivers very high, single fractions of daily radiation up to five total fractions, has been termed stereotactic body radiotherapy (SBRT). Challenges in using external beam radiotherapy for HCC are many, with successes being realized using image-guided radiotherapy (IGRT) to assist in the delivery of 3DCRT, IMRT, and SBRT, along with respiratory motion compensation and tumor visualization [81, 86]. The use of proton beam radiotherapy represents a different type of energy than photons that, by physical characteristics, can achieve superior dose deposition compared to 3DCRT [87, 88].

### Indications for External Beam Radiotherapy (RT) in HCC

Radiotherapy (RT) can have a meaningful role in all stages of HCC—with a proven ability to sterilize tumors similarly to other local ablative approaches like RFA [89]. In the BCLC classification, Stage 0 and early stage A patients who cannot undergo surgical resection, transplant, or RFA are candidates for curative intent radiation therapy. Potentially transplantable patients can benefit from RT as a bridge to transplant while on the wait list. In stage B and C, RT has efficacy in situations where TACE has been ineffective or is unsuitable. This is particularly important in patients with portal vein invasion where TACE is contraindicated, and where TARE may not be possible or ineffective [81, 86].

### Stereotactic Body Radiotherapy (SBRT) for HCC

There is strong interest in pursuing SBRT for HCC due to the increased ability to spare normal liver tissue from receiving tolerance doses of radiation. Four prospective studies and four retrospective reports are available from 2006–2011. These involve a range of 8–60 patients and are limited to single institutions. Despite the lack of larger, randomized, controlled data, the positive outcomes in all stages of HCC are proven with a wide array of fraction sizes and total doses. Excluding the one study that had only eight patients, the remaining three used at least five different fractionation schedules adjusted for Child-Pugh A or B classes. One-year survival ranged from 48–79 % in these heterogeneous groups [90–92].

### Proton Beam Therapy (PBT) for HCC

Because of increased control of radiation dose deposition at any depth in the body, there has been intense interest in using PBT for HCC [93]. Prospective studies to date using PBT have been positive regarding toxicity and tumor control, with encouraging overall survival rates in selected HCC patient groups in Eastern and Western populations [94–97]. Compared to SBRT, there are more prospective studies (ten), with each study reporting greater numbers of patients, ranging from 76 to 318 patients. It is not known whether SBRT or PBT is superior or equivalent in outcomes of HCC patients. Likely, they will be complementary to each other based on factors such as tumor size, distribution, and location in the liver. Dawson has suggested that photon beams (3DCRT, IMRT, SBRT) might be best employed in Child-Pugh A patients with tumors in the right lobe near the dome, less than 6 cm in size. Protons may be utilized best in Child-Pugh B, tumors larger than 8 cm, and those central/medial in the liver [81, 93]. There is only level 2a evidence supporting any form of radiation in HCC, however, combined with the retrospective reports of hundreds of patients, there is a significant weight of evidence supporting radiotherapy in all stages of HCC [87, 88].

## Conclusions

Hepatocellular carcinoma patients who cannot receive curative approaches can derive significant benefit in quality of life and survival if they are eligible for the intraarterial or external therapies presented. New technologies exploiting both approaches are being tested clinically, including external radiation using carbon ion beams, combined chemotherapy and TARE, and variations on TACE, both mechanically and via the chemotherapy agent deployed.

It should be recognized that TACE is a heterogeneous group of procedures in terms of materials, extent and selectivity of vessel occlusion, and timing of repeated sessions. Good tumor responses are generally observed when a small number of small tumors are embolized in a selective fashion (ideally through a distinct feeding vessel). Based on two positive clinical trials and three meta-analyses, TACE is the standard of care for HCC patients in the intermediate stage. DEB-TACE has recently become a more standardized way of performing TACE with similar outcomes. Y90-RE is a form of brachytherapy for liver tumors in which the source of radiation has to access the network of tumor neovascular plexi. In contrast with TACE, evidence supporting the use of TARE in the treatment of HCC patients comes from consistent, large-cohort series involving patients with more advanced HCC, who are not suitable for other locoregional therapies, or who have failed treatment with TACE.

TACE and TARE should not be considered competing therapies, but rather complementary tools. For those patients with small to medium-sized tumors that can be treated selectively, TACE can be provided in most centers. TARE could be an alternative to repeated TACE for patients that fail to respond to initial TACE, and a first option in those who are poor candidates to TACE, mainly because of bulky disease and PVT, but still have a good liver function. The results of ongoing clinical trials will soon establish whether sorafenib or other targeted therapies improve the outcome of patients treated by TACE and TARE.
